# Cadaver Lab Education in Surgical Residency

**DOI:** 10.7759/cureus.112596

**Published:** 2026-07-13

**Authors:** Ben Kaplan, Tomer Vaintraub, Dor Yakoby, Joseph Palatchi Oldak

**Affiliations:** 1 Orthopedics, Rambam Medical Center, Haifa, ISR; 2 Orthopedic Surgery, Rambam Medical Center, Haifa, ISR; 3 Medicine, Universidad Anahuac, Mexico City, MEX; 4 Orthopedic Surgery, Hospital Angeles Universidad, Mexico City, MEX; 5 Orthopedics and Traumatology, Hospital Infantil Privado, Mexico City, MEX

**Keywords:** anatomy and physiology, medical residency, ortho surgery, physician training, simulation in medical education

## Abstract

Cadaver labs are a unique and invaluable resource, providing medical students with a high-level learning environment for developing anatomical knowledge and achieving an exceptional level of proficiency. These labs are typically maintained by medical schools exclusively for the purpose of teaching medical students. In contrast, surgical residents often lack access to cadaver labs, despite their critical need to develop advanced anatomical understanding and manual skills essential for daily clinical practice. In this opinion article, we propose expanding the use of cadaver labs to include routine integration within surgical residency training programs. We present a framework that incorporates cadaver lab training throughout different stages of residency, from specialty-specific anatomy education to advanced procedural and surgical rehearsal. We also highlight their potential to facilitate surgical innovation and research. We argue that this broader integration could significantly enhance surgical education while maximizing the educational and scientific value of these costly resources.

## Editorial

Introduction

In many institutions around the world, cadaveric dissections are an integral and mandatory part of formal anatomy training in medical schools. Dissections allow hands-on experience and an opportunity to achieve profound anatomical knowledge and attention to anatomical subtleties. While virtual reality tools and 3D software are gaining popularity and, in some cases, replacing cadaveric dissection, they are currently not able to fully replicate the visual and tactile complexity of human cadavers. Therefore, cadaveric dissection remains a highly common teaching method despite the high costs of establishing and maintaining a cadaver lab. Cadaver labs are usually sustained by medical schools for the single purpose of teaching anatomy to medical students in their early years of medical education. However, we believe that cadaver labs should be utilized more broadly, extending their role beyond foundational anatomy training to include advanced resident education, where specialty-specific anatomy instruction can be combined with surgical and procedural rehearsal.

Several years after taking formal anatomy training and performing cadaveric dissection, graduating medical students who choose a surgical specialty are at a point in time in their careers where they need to develop a high proficiency in anatomy beyond the basic understanding of medical students. However, this needs to be achieved with fewer teaching resources, particularly in many institutions, without regular access to a cadaver lab [1]. In this paper, we examine the role of cadaver-based education in surgical residency and propose a structured framework for its integration into modern surgical training programs.

Achieving anatomical proficiency for training surgeons

Anatomy is one of the key fundamentals that a training surgeon needs to harness. While textbooks and other anatomical resources often simplify anatomy for clarity, surgeons must develop a deep familiarity with human anatomy, along with its inherent complexities. Cadaver dissections provide an opportunity to study anatomy in detail, allowing residents to observe intricate anatomical relationships and subtleties that may not be critical for medical students but are of significant importance for surgeons. In orthopedic surgery, for example, cadaver-based training enables detailed visualization of joint anatomy, fracture planes, ligamentous structures, and surgical approaches that are often only partially visible during live procedures. Additionally, the hands-on process of dissecting cadavers and exposing anatomical structures might enhance memory retention and assist in memorizing the anatomy. While participating in surgical procedures enables residents to witness anatomy in a clinical context, surgeries often involve small incisions, leaving much of the relevant anatomy unexposed. Cadaver dissections, on the other hand, could allow residents to gain a complete understanding of anatomy, including structures that may never be visible during surgery.

Several studies have highlighted the benefits of studying anatomy through cadaver dissections. For instance, Kumar et al. investigated the impact of using cadavers to teach facial anatomy to aesthetic physicians and demonstrated that even a short, two-day cadaver lab training significantly enhanced anatomical knowledge and comprehension measured by pre-course and post-course evaluation [2]. Similarly, Jeyakumar et al. used a questionnaire to examine the perceptions of medical students regarding cadaveric dissections. They found that the majority of students agreed that dissections facilitate active learning, deepen their understanding of anatomy, and help consolidate their knowledge [3]. Additionally, the study highlighted that performing dissections significantly contributed to the development of hand-eye coordination and manual dexterity, further enhancing the student's learning experience.

Cadaveric dissection as a part of medical training is usually carried out in the early years of medical school, most often in the first year. This generates a number of limitations and concerns for training surgical residents. Importantly, a significant time gap frequently exists between undergraduate anatomy training and the start of surgical residency. During this prolonged period, much of the anatomical knowledge is forgotten. Furthermore, during formal anatomy training, medical students are often uncertain about which specialty they will ultimately pursue. As a result, they may undervalue the importance of studying specific anatomical systems that could later prove crucial for their chosen specialty. Currently, the best resources for studying anatomy, including access to expensive cadaver labs, are provided during the early stages of medical training. However, we argue that for surgeons, these resources are most essential during the early stages of residency, after they choose a specialty, and as daily practice demands a deep and comprehensive understanding of anatomy.

Developing surgical skills and confidence

While medical schools provide students with exposure to surgical specialties and allow some participation in surgical procedures, this is by no means an adequate preparation for the technical aspects of a surgical residency [4,5]. Residents are required to develop various technical skills and eventually learn to perform full surgical procedures. The long process of technical development mainly occurs in the operating room, slowly gaining more autonomy and experience during real procedures on live patients. While surgical simulators, virtual reality solutions, and 3D tools may assist in resident training and provide important advantages, such as repetition and accessibility, they are not able to replicate the visual complexity of a real human body. In contrast, cadaver labs can offer the opportunity to not only study anatomy but also to learn and practice surgical techniques and procedures in a safe environment without risking the patient.

The cadaver lab could provide an ideal environment for developing specific surgical skills, such as suturing, inserting orthopedic wires, handling a saw, or manipulating various types of endoscopes. Repeated practice of these skills could help residents improve their precision and confidence before entering the operating room. As residents progress in their training and gain more autonomy in the operating room, the cadaver lab could then be used to practice specific parts of surgical procedures, such as the surgical approach and exposure-tasks that often require only standard tools readily available in the cadaver lab. Furthermore, it provides an invaluable opportunity to practice complex regional approaches-such as posterior approaches to the hip, deep exposures of the pelvis, or intra-articular joint lines-where a precise, three-dimensional grasp of neurovascular bundles is critical to preventing catastrophic intraoperative complications. In the later years of residency, the lab could also be utilized to perform full surgical procedures.

Cadaver lab training for surgical residents is not mandatory in most institutions; however, it is sometimes accessible through external courses or as part of the curricula in certain programs. Consequently, some studies have been published on its effectiveness. For example, Lewis et al. described a cadaver-based training program for general surgery residents using fresh-frozen cadavers [6]. Beyond anatomical demonstrations, these sessions allowed residents to practice surgical techniques such as suturing, chest tube insertions, and other procedures. A survey conducted to evaluate the contribution of these sessions found that residents perceived them as highly effective, with most expressing a desire to participate in additional sessions. In another example, vascular surgery operating skills were practiced by residents on cadavers. This training was highly valued by participants, with the vast majority (97.8%) giving a perfect evaluation score and recommending these sessions to other residents [7].

Research opportunities

Providing residents with adequate access to cadaver labs could also offer new opportunities for research and academic experience. For example, using human cadavers, anatomical studies could be conducted to describe important anatomical variations. These variations could be described using dissection and histology but also with advanced imaging tools such as micro-CT and high-resolution MRI that currently cannot be applied to living patients [8]. Furthermore, various biomechanical studies could be conducted on tendons and ligaments that may have an important impact on clinical practice. Novel surgical approaches could also be developed, and studies evaluating new surgical technologies could be conducted. Finally, the impact of cadaver-based training on resident development could be examined more rigorously from an academic standpoint.

Proposed framework for integrating cadaver training into residency 

To maximize its educational value, cadaver-based training should be systematically incorporated into surgical residency through a structured, stage-based approach aligned with the progressive development of surgical competencies. Rather than being offered as sporadic or optional sessions, cadaver laboratory exposure should be integrated longitudinally throughout residency, with clearly defined objectives tailored to each stage of training. During the early years of residency (postgraduate years one to two), cadaver-based sessions should primarily focus on reinforcing anatomical knowledge in a clinically relevant context. At this stage, residents would benefit from revisiting key anatomical regions related to their chosen specialty, with an emphasis on spatial orientation and identification of critical structures. In addition, these sessions can introduce fundamental surgical concepts, including basic approaches, tissue handling, and familiarity with standard instruments.

As residents progress to intermediate stages of training (postgraduate years three to four), cadaver-based education should shift toward procedure-oriented learning. At this level, sessions can be designed to simulate specific surgical interventions relevant to the specialty, allowing residents to practice operative steps in a controlled environment. The focus should be on developing technical proficiency, understanding procedural workflows, and refining hand-eye coordination. In the advanced stages of residency (postgraduate year five and beyond), cadaver laboratories can serve as a platform for performing complete surgical procedures and managing complex scenarios. At this level, training may include advanced techniques, complication management, and even preoperative rehearsal of challenging cases. Such practice enables residents to consolidate their technical and cognitive skills before performing similar procedures in the operating room, thereby enhancing both confidence and patient safety. To achieve its full potential, cadaver-based sessions should be conducted at regular intervals throughout residency. Each session should include clearly defined learning objectives and structured supervision by experienced faculty who can provide suitable feedback.

Overcoming current challenges

The costs and resources required to sustain a cadaver lab pose a major challenge in incorporating cadaver-based training in residency. Establishing and maintaining a cadaver lab can be extremely costly, and currently, many surgical residents do not have frequent access to these facilities. In our opinion, this issue could be addressed in many institutions worldwide through collaboration between anatomy departments in medical schools, many of which maintain a cadaver lab and surgical departments.

We believe that collaboration between anatomy and surgical departments could expand the educational value of cadaver labs beyond teaching anatomy to medical students. This partnership could facilitate surgical training for resident doctors, providing them with much-needed access to cadaver labs. Such collaboration is technically feasible, as performing surgical procedures on cadavers causes minimal harm, allowing medical students to continue performing full dissections. Although most cadavers in medical schools are preserved in formaldehyde, which alters their mechanical properties compared to living tissue, in our experience, they still simulate the handling of live human tissue sufficiently well. However, to mitigate the limitations of tissue rigidity inherent to formalin fixation, institutions could consider incorporating a subset of fresh-frozen or Thiel-soft-preserved cadavers specifically designated for procedure-oriented training [9]. Finally, this collaboration could benefit medical schools by fostering opportunities for joint research with surgical departments, promoting scholarly productivity and innovation in surgical education, increasing medical students' exposure to surgical specialties, facilitating mentorship and early career exploration, and enhancing the overall impact of medical schools on medical education by better integrating basic science and clinical training.

Conclusion

Incorporating cadaveric dissection into surgical residency programs could have a significant impact on enhancing anatomical knowledge and technical skills. Currently, cadaver labs in medical schools are primarily used to teach medical students. However, surgical residents, who require a deeper and more nuanced understanding of anatomy, often lack regular access to these facilities. As a result, cadaver labs are not fully utilized to their fullest potential in advancing medical education. Incorporating structured training for surgical residents in cadaver labs could not only enhance the quality of residency training but also broaden the educational impact and value of cadaver labs within medical schools.

## References

[1] Stengel FC, Bozinov O, Stienen MN (2022). Transformation of practical exercise in neurosurgery depending on the level of training. Brain Spine.

[2] Kumar N, Rahman E (2017). Effectiveness of teaching facial anatomy through cadaver dissection on aesthetic physicians' knowledge. Adv Med Educ Pract.

[3] Jeyakumar A, Dissanayake B, Dissabandara L (2020). Dissection in the modern medical curriculum: an exploration into student perception and adaptions for the future. Anat Sci Educ.

[4] (2014). Statement on surgical preresidency preparatory courses. Ann Surg.

[5] Grbic D, Dent DL, Jurich DP, Roskovensky L, Andriole DA (2026). US medical school graduate readiness for the transition to surgical residency: a national study. J Am Coll Surg.

[6] Lewis CE, Peacock WJ, Tillou A, Hines OJ, Hiatt JR (2012). A novel cadaver-based educational program in general surgery training. J Surg Educ.

[7] Reed AB, Crafton C, Giglia JS, Hutto JD (2009). Back to basics: use of fresh cadavers in vascular surgery training. Surgery.

[8] Kim S, Sakaie K, Blümcke I, Jones S, Lowe MJ (2021). Whole-brain, ultra-high spatial resolution ex vivo MRI with off-the-shelf components. Magn Reson Imaging.

[9] Kovacs G, Levitan R, Sandeski R (2018). Clinical cadavers as a simulation resource for procedural learning. AEM Educ Train.

